# Facilitators and inhibitors of traumatic brain injury transfers: A fieldwork investigation

**DOI:** 10.1002/nop2.1874

**Published:** 2023-06-14

**Authors:** Karin Højbjerg, Ingrid Poulsen, Ingrid Egerod

**Affiliations:** ^1^ Department of Learning and Philosophy Aalborg University Copenhagen Copenhagen Denmark; ^2^ Department of Brain Injury, Copenhagen University Hospital Rigshospitalet Copenhagen Denmark; ^3^ Department of Nursing Science, Health Aarhus University Aarhus Denmark; ^4^ Department of Intensive Care, Copenhagen University Hospital Rigshospitalet Copenhagen Denmark; ^5^ Department of Clinical Medicine University of Copenhagen Copenhagen Denmark; ^6^ Present address: Department of Nursing and Nutrition, Faculty of Health Copenhagen University College Copenhagen N Denmark; ^7^ Present address: Department of Clinical Research Copenhagen University Hospital, Amager and Hvidovre Hvidovre Denmark

**Keywords:** decision‐making, health transitions, nursing, patient transfer, qualitative research, traumatic brain injury

## Abstract

**Aim:**

Intensified healthcare specialization has increased the need for patient transfers. We aimed to describe in‐hospital and interhospital patient transfer decisions during the traumatic brain injury (TBI) trajectory from a nursing perspective.

**Design:**

Ethnographic fieldwork.

**Methods:**

We used participant observation and interviews at three sites representing the acute, subacute and stable stages of the TBI trajectory. Deductive analysis was applied supported by transition theory.

**Results:**

During the acute stage (neurointensive care), transfer decisions were facilitated by physicians assisted by critical care nurses, in the subacute stage (highly specialized rehabilitation), transfer decisions were collaborative among in‐house healthcare professionals, community staff and family, and during the stable stage (municipal rehabilitation), transfer decisions were made by non‐clinical staff. Most of the resources allocated during the trajectory went towards highly specialized rehabilitation, whereas more resources are needed during the end of the trajectory.

**No Patient or Public Contribution:**

Patients and the public were not involved in this study .

## 1 INTRODUCTION

Patient transfer is common in contemporary healthcare systems as sub‐specialization encourages continuous movement of patients towards the most appropriate department for treatment. Patient transfer may be motivated by incentive structures such as diagnosis‐related groups, where professional activity is determined by cost, quality and effectiveness (Busse et al., 2011). Alternative incentives have been suggested to improve patient outcomes, such as patient‐centred care (Editorial, 2018). Patient transfer between hospitals puts high acuity patients at risk of poorer outcomes, depending on the time of transfer (Mueller, Fiskio, & Schnipper, 2019). Moreover, patient transfer negatively impacts nurses' workload (Blay et al., 2017). Interhospital transfer has been associated with higher cost, longer hospital stays and discharge delays due to the discontinuity of care (Mueller, Zheng, et al., 2019). Inter‐ and intra‐hospital patient transfers have increased globally as hospitals attempt operate near full capacity (Blay et al., 2017). In this study, we focus on transfer practices in relation to patients with traumatic brain injury (TBI), as this vulnerable group of patients is often subject to multiple transfers and transitions (Ismail et al., 2020; Picetti et al., 2013).

## 2 BACKGROUND

Studies are needed that describe the care‐transition burden related to patients with TBI. To this end, a large European cohort study recently identified common transition pathways in TBI‐trajectories (Borgen et al., 2021). Many identified pathways progress from the intensive care unit (ICU) to the ward, and finally to the post‐acute discharge destination, such as home or care facility at the end of the trajectory. Most transitions take place in the ICU stratum (Borgen et al., 2021). Moreover, patients with severe TBI experience more transfers than patients with less severe injury.

Patient transfers increase the number of transitions patients experience during their illness trajectory. The transition model developed by Meleis et al. shows that transitions are influenced by variables, such as transition type, conditions (facilitators and inhibitors) and patterns of response (Meleis et al., 2000). Transitions are described as periods of change and instability between relatively stable periods. The theory further classifies facilitators and inhibitors as personal, interpersonal, organizational, communal and societal (Chao et al., 2020). Studies are emerging showing that not only the patient suffers during multiple transitions, but also the family members as well (Karlsson et al., 2020; Masterson & Brenner, 2016; Oh et al., 2015). A recent qualitative study identified discharge facilitators after TBI rehabilitation such as adequate patient health, access to healthcare services, availability of family caregiver, attachment to home, commitment to loved one, possibility of transitional phase (e.g. going home on weekends), and patient and family collaboration (Souesme et al., 2022). Importantly, the particular needs of patients evolve through the trajectory as their health improves, suggesting that professional specialization should change accordingly.

Our study was conducted in Denmark, where healthcare is financed through taxes without direct payment. The Danish Health Care Act states that all citizens should have easy and equal access to healthcare services (Sundhedsloven, 2008). The healthcare system is organized in two sectors, each with its own budget (primary and secondary healthcare sectors) and regulatory framework, which might impact patient transfer across sectors.

In our previous study, we chose a dispositional and relational approach (Bourdieu, 2018) in combination with a profession perspective (Abbott, 2005) to the data material focusing on how durable and often underrated habits of thought and action might structure situated interaction. These embodied professional and organizational relations formed decisions among the professionals with a risk of contributing to inequality in health if patients were transferred for other reasons than their immediate health, for example if physicians transferred a patient for strategic reasons (Højbjerg et al., 2019). In the present study, based on a secondary analysis, we take a situationist approach, where the situation determines the possibilities for action. It is our assumption that nurses might act as buffers in this system and that awareness of facilitators and inhibitors of transfer might ease the transition. The aim of our study was to describe in‐hospital and interhospital patient transfer decisions during the TBI trajectory from a nursing perspective. We wished to gain more knowledge about facilitators and inhibitors to patient transfer during the TBI treatment and rehabilitation trajectories.

## 3 METHODS

The methodological orientation of our study was ethnographic fieldwork applying participant observation, in‐situ interviews and fieldnotes generated during fieldwork (Hammersley & Atkinson, 2007; Walford, 2009). The present study is a secondary analysis of a study published in 2019 in an anthology of ongoing social policy changes in relation to disability and rehabilitation (Højbjerg et al., 2019). The present study takes a clinical and nursing view of issues described in the previous study.

### 3.1 Sites and setting

The treatment and rehabilitation trajectory for patients with moderate and severe acute brain injuries in Denmark can be described in three stages: the acute and subacute stages at public hospitals in the secondary healthcare sector, and the stable stage within the primary healthcare sector. Acute TBI treatment is offered at the four most specialized hospitals in the country, whereas highly specialized rehabilitation at the subacute stage is managed at two national centres, east and west (Engberg, 2007). Rehabilitation at the stable stage is offered at the municipal level closer to the patients' homes. We selected the sites to represent the three stages: the acute stage (Site A), describing initial hospitalization and intensive care; the subacute stage (Site B), describing highly specialized rehabilitation; and the stable stage (Site C), describing long‐term rehabilitation at the municipal level. Sites A and B were university hospitals selected for their high level of specialization. Site C was selected to represent the final stage of the trajectory supporting patients with multiple and complex physical and psychosocial issues.

### 3.2 Data collection

The participants included individuals present during participant observation and in‐situ interviews: Registered Nurses, vocational nurses, physicians, physiotherapists, occupational therapists, managers, patient and family. Participants were contacted by the management at each site and were selected as a convenience sample. We completed data collection in 2018, focusing on formal and informal interprofessional meetings at three sites, where decisions were assumed to be made regarding patient transfers. Initially, we visited each site for 3 days to get an idea of the organization of the daily work and transfer practices. Our attention was on the physical setting, actors, action and interaction in relation to patient transfer. Fieldwork at Sites A and C was conducted as non‐participatory observation of institutional activities for 5 days at each site, and at site B as non‐participatory observation of interdisciplinary meetings and staff working with the patients. We conducted informal in‐situ interviews based on observations in relation to patient transfer.

Fieldwork at Site A was oriented towards physicians that were formally in charge and the coordinating nurse. Nurses and a physiotherapist were observed as collaborators in the decision process. At Site B, observations focused on activities and decision‐making during interdisciplinary meetings. Ten scheduled meetings were observed focusing on the dominant argument for patient transfer. Each meeting involved 8–12 participants, including staff and family caregivers. A physician, a nurse and an occupational therapist were observed during their work with severely brain‐injured patients. The fieldwork at Site C focused mainly on vocational nurses as the daily healthcare providers involved in preparing the resident for transfer and arranging discharge to home or permanent care. All told, data were collected over the course of 19 days, during which 21 in‐situ interviews were conducted (Table 1). The first author (KH) spent 135 h conducting ethnographic fieldwork, and a student, not involved in the study, transcribed the fieldnotes and in‐situ interviews.

**TABLE 1:** Distribution of in‐situ interviews (*n* = 21).

Site	Informants by profession	Number of interviews (*n* = 21)
Site A (*n* = 7)	Senior physicians (anaesthesiologists)	4
	Clinic manager	1
	Coordinating Registered Nurse	1
	Senior physician at interim unit	1
Site B (*n* = 5)	Senior physician	2
	Registered Nurse	1
	Physiotherapist	1
	Occupational therapist	1
Site C (*n* = 9)	Managers	2
	Vocational nurses	3
	Lead volunteer	1
	Local case manager	1
	Local case coordinator	1
	Occupational therapist	1

### 3.3 Data analysis

The research team consisted of three female Ph.D.‐prepared nurses with experience in qualitative research. Two had experience with patients with TBI, but none of the investigators had prior knowledge of the actual participants. The first author (KH) performed the initial coding of the data and rest of the team participated in critically discussing the best interpretation of the findings. Ethnography has been described as the art and science of describing a group or culture (Walford, 2009). As such, the field observer set out to uncover the particular cultures of the three sites in the study. During analysis we focused our investigation at the organizational level, people and places and situations (Jerolmack & Khan, 2017). After reading and re‐reading the fieldnotes and interview transcripts, we constructed an analytical matrix and analysed deductively according to the trajectory stages: acute, subacute and stable. The transition theory includes the concept of transition conditions identified during data analysis (Chao et al., 2020). Within each stage, we analysed according to the transition conditions, facilitators and inhibitors, which could be classified as personal, interpersonal, organizational, communal or societal, Table 2. We performed narrative description of our findings and used investigator triangulation throughout the process to confirm and contest each other's views.

**TABLE 2:** Analytical matrix.

Analysis according to treatment stage	Identification of transition conditions: Facilitators and inhibitors of patient transfer
Acute stage	*Facilitators*: Interhospital transfers depended on mutual understanding and collaboration among physicians across the hospitals in the region (interpersonal facilitators). Transfer decisions were an integral part of daily practice and the united focus at daily meetings. Registered Nurses assisted in decision‐making (personal facilitators)
	*Inhibitors*: Capacity constraints and the physicians' inability to predict and set limits for the number of admissions (organizational inhibitors)
Subacute stage	*Facilitators*: Meetings had a tight structure with input from healthcare professionals and family caregivers (interpersonal facilitators). Agreement was facilitated by knowledge sharing among members of the in‐house team and community staff. A collaborative culture and spirit of negotiation strengthened collaboration as the basis of decision‐making (communal facilitators)
	*Inhibitors*: The potentially asymmetrical relationship (in terms of number and knowledge) between staff and family might have intimidated family caregivers during decisional meetings (societal inhibitors)
Stable stage	*Facilitators*: The final transfer home or to a care facility was enabled by vocational nurses that were trained in the practical aspects of social and health care based on patient‐centred values (personal facilitators)
	*Inhibitors*: Transfer was inhibited by the mismatch between the increasing complexity of the residents' needs and the vocational nurses' lack of decisional capacity (communal inhibitors). This was combined with multiple and often absent community staff and excessive bureaucracy (societal inhibitors)

### 3.4 Ethical considerations

The study was performed in accordance with the Declaration of Helsinki (2013). Written informed consent was obtained and participants were informed about anonymity and confidentiality with the option to withdraw from the study at any time. No personal data were used in this study. The study was approved by management at the three study sites, but we were not required to obtain further Research Ethics Committee approval as there is no review board for qualitative studies in Denmark.

## 4 FINDINGS

### 4.1 Site A: The acute stage, neurointensive care. Transfer practices as collaborative, mostly monodisciplinary and adhering to structural conditions

Initial treatment was offered at the main referral hospital for brain injuries (Site A). Severely injured patients were admitted through the trauma centre. The core trauma team consisted of sixteen healthcare professionals providing life‐saving and stabilizing treatment before patient transfer to surgery or one of six ICUs at the hospital. The team consisted of specialized physicians, Registered Nurses, physiotherapists, X‐ray technicians and other core staff. Patients with severe TBI were transferred to the neurointensive care unit (neuro‐ICU). All admitted patients were diagnosed and treated, regardless of age and physical condition. This was described the ‘open house policy’, recently implemented. The Clinic manager explained:

Before, we admitted patients with fewer diagnoses, but it was decided by the top management that we should admit patients with a wider range of brain injuries. This includes full treatment of older patients with strokes. We have expanded the unit from 14 to 20 beds, but it is still not enough (Clinic manager).

The current bed shortage also impacted the receiving departments, putting pressure on the physicians to maintain patient flow. The pressure was evident when physicians and the coordinating nurse met to discuss patient transfer status, being the main issue at morning and noon conferences. When an acute bed was needed in the neuro‐ICU, other patients might have to transfer prematurely. If a patient was not ready for highly specialized rehabilitation (Site B) or if Site B was full, the patient had to make a ‘detour’ to an ‘interim unit’ or hospital, entailing more patient transfers and transitions. Transfers were unavoidable, even after capacity increases in the region. Although every transfer presented a risk, the procedure was usually performed with mutual understanding among the regional physicians.

Well, I think it has something to do with … other than wanting to help each other … that we know each other very well among the intensive care units. It's a small sub‐specialty, and I often know their [the physicians'] names. And we all know the problems of overcrowding, the next day I might be in the same situation (Anaesthesiologist at an interim unit).

The ‘open house policy’ combined with the limited number of available beds, was a driving factor in transfer practices, giving little room for optimal transfer planning.

We are all aware of these problems, any ICU fears vacant beds. So, we are all familiar with situations where patients are transferred prematurely. There are not enough available beds … we work together to help solve these situations (Head physician at Site A).

One head physician described patient transfers as a ‘logistic hell’, indicating that more options should be explored. The coordinating nurse provided patient data and an overview of options at the neuro‐ICU and the potential receiving units. The detour to an interim unit or hospital was only considered if the appropriate treatment was available, but an incoming patient put pressure on the system at all sites. Patient transfer could have a domino effect. Each hospital in the region had specific sub‐specialties and it was important to transfer the patient to the most appropriate hospital to match the main diagnosis.

In summary, the hospital transfer puzzle required expert medical knowledge and was delegated to physicians with authority to admit and discharge patients. Nurses and physiotherapists supported the decisions by informing physicians of progress or changes. Table 2 shows the facilitators and inhibitors of patient transfer at Site A.

### 4.2 Site B: The subacute stage, highly specialized neurorehabilitation. Transfer practices as interprofessional negotiation with family involvement

The organization of meetings at the subacute stage differed from the acute stage. Specialist physicians were replaced by a collaborative team of healthcare professionals (e.g. physicians, neuropsychologists, physiotherapists, occupational therapists, speech and language pathologists, social workers, Registered Nurses and vocational nurses). Transfer decisions were made at ‘treatment meetings’ or ‘team meetings’.

*Treatment meetings* were held every 2 weeks with the collaborative team, family caregivers and, on occasion, patients. Rehabilitation plans were reassessed as each staff member reported on patient progress within their specialty area. An example from the fieldnotes:

Physician: “What is the patient's level of activity?”
Physiotherapist: “Well, we have tilted him. He is stable, but he can't participate from a low position. He can't put weight on his feet, and I haven't seen him take initiatives.”
Registered Nurse: “I have seen him respond to a verbal request, I mean bend and stretch both legs – strongest on the left of course, but he only responds when it is requested.” (Fieldnote A: treatment meeting)

The physician summed up the assessment, and the rehabilitation plan was adjusted, including readiness for transfer. Although the meeting was led by the physician, transfer decisions were mostly collaborative using input from each participant.

*Team meetings* were held every 6 weeks with the collaborative team to assess readiness for transfer or discharge. Each meeting lasted approximately 1 h. The physician made the initial decision on patient transfer, but after a round where the professional team contributed in turn with their views, the decision could be changed if there was a stronger argument. Up to seven in‐house staff, three community staff (community coordinator, brain injury coordinator and homecare coordinator), and two family caregivers were usually present. Family caregivers could argue for an extension of six more weeks in rehabilitation. In two cases of the ten observed, family caregivers convinced the team to grant the patient such an extension. An example from fieldnotes:

Mother: “But what are you talking about? Being violent? [to the physician] I don't understand – he has always been nice and calm”.
Physician: “It's because of the brain damage. It's not because he is violent.”
Mother: “Being able to come down to the smoking area when he wants to, calms him down. I told you already before he came here, that this is necessary. If you had let him smoke when I asked you, he wouldn't have been so agitated” (Fieldnote B: team meeting).

If the patient was ready for transfer, the community staff joined the meeting after the transfer decision was made. The objective was to decide on the best location (Site C) according to the patient's needs. The local municipality had the practical and financial responsibility for this stage of rehabilitation.

In summary, the subacute stage showed a higher degree of interdisciplinary collaboration and family involvement in decision‐making than the acute stage. Decisions were made at longer intervals that at the acute stage. Patients were transferred when they were ready, according to the team or transfer was postponed at the request of the family. Table 2 shows the facilitators and inhibitors of patient transfer at Site B.

### 4.3 Site C: The stable stage. Transfer practices based on patient‐centred values and bureaucratic standards of external collaborators

Site C was a self‐governed communal rehabilitation centre representing the final stage before discharge or transfer to home or permanent care. The identity of the patients changed to ‘resident’, signalling that their needs had shifted from health‐related to bio‐psycho‐social and economic issues. Patient‐centred care (the term used) was the declared value of this three‐story institution, where one floor was dedicated to residents with alcohol or drug abuse. Residents with brain injury shared the remaining two floors with residents with mixed health and socioeconomic issues.

Our main focus is to speak for those who are unable to speak up for themselves … we take the side of the resident and … act as a watchdog … acknowledging the resident's feelings (Manager).

The in‐house staff consisted of a manager, co‐manager, vocational nurses, former residents, an occupational therapist and volunteers. The two managers were master's prepared in sociology and psychology. The vocational nurses had a 2‐year diploma in social work and health care. The institution did not employ Registered Nurses or physicians. The general practitioner (GP) was contacted if medical attention was required. Both staff and management expressed frustration as they often experienced the GPs as unavailable and vocational nurses interpreted this as lack of interest:

One day, a vocational nurse discovered that a resident was responding poorly. The resident had multiple diagnoses, including diabetes, liver dysfunction, and symptoms following brain injury. The vocational nurse also observed that the resident's level of consciousness had noticeably changed. She contacted the GP immediately, but he did not come before the next day. As it turned out, the patient had pneumonia and was admitted to the hospital for observation. The vocational nurse stated: ‘His GP doesn't really want to have anything to do with him’. (Fieldnote C).

Site C did not offer the same medical availability as seen at the previous sites. The focus was on a broader spectrum of bio‐psycho‐social complexity, for example housing, income, social network and work relations (if any). Many residents were digitally illiterate and unable to manage their credit cards and computer codes. Vocational nurses were the frontline staff to assist the residents, but they were not systematically present at the monthly meetings with the managers and the communal coordinator to make discharge decisions. The manager described the meetings with the communal coordinator as ‘superficial’ and ‘goal‐oriented’, often resulting in discussions about payment rules and municipal responsibility. As a result, discharge was often delayed. The daily staff experienced a lack of understanding of the resident's situation and chances of discharge. Vocational nurses were distressed by the way the residents were neglected and tried to speed up the final arrangements for transfer with external collaborators, that is by means of arranging home visits, by providing additional information, requesting updates via email or scheduling extra meetings with a communal coordinator. The distress was compounded by lack of collaboration with communal staff, for example a physiotherapist failing to show up at a home visit. The following is an excerpt from an in‐situ interview with a vocational nurse:

“So, you know, Stan (a resident) should have received a letter within two weeks … And this is the problem!” I tell Bob (the social worker in charge): “Stan is suffering from amnesia, he forgets stuff, he can't do things.” Then Bob says: “But I sent it! Maybe I sent it electronically!” So, I think to myself: “What is this idiot thinking? Stan has been evicted. He has no credit card. He gets a new credit card every week because he forgets where he puts it. He hasn't got a social security card … He doesn't even open his mail … And then they send an email?” I say: “Very clever … really … he doesn't even have a wallet”. Bob works with this kind of residents, and I think: “Can't he even put two and two together?” I get pretty upset by these cases …” (Vocational nurse).

In summary, the distance between transfer decision and execution was greater at Site C than Sites A and B. Many decisions were de facto left to the vocational nurses who were not in a position to manage the complexity of residents' many needs through the municipal bureaucracy. Vocational nurses were without medical and socioeconomic authority, and, though supported by managers, they lacked the power to execute decisions effectively. The social workers and GPs were not integrated in the daily care and joint transfer decisions. Table 2 shows the facilitators and inhibitors of patient transfer at Site C.

### 4.4 Cost of care through the trajectory

Finally, we wished to compare the cost at each of the three sites to describe allocation of resources and configuration of services. The daily cost per patient was: Site A = EUR 3500, Site B = EUR 2000 and Site C = EUR 220. We obtained the numbers from each site to ad information at the organizational level. As expected, the daily price of treatment and care decreases substantially through the illness trajectory. Site A offered the highest medical expertise measured in terms of education, experience and staffing. Site B offered a high degree of interdisciplinary specialization distributed among a larger number of healthcare professionals with different educational backgrounds. Site C offered little specialization and used staff with inadequate authority as the main care personnel. Conversely, if we look at the actual length of stay for many patients: 1 week at Site A = EUR 24,500, 6 weeks at Site B = EUR 84,000 and 3 months at Site C = EUR 19,800. In this perspective, highly specialized rehabilitation is the costliest per patient, which corresponds to the many specialists involved at this important stage.

## 5 DISCUSSION

Our main finding was that transfer decisions were made on a very different basis at the three sites investigated. During the acute stage decisions were made on a moment‐to‐moment basis to accommodate the need for acute beds in the region vis‐à‐vis the present patient's needs. At this stage there was a risk of premature transfer. During the subacute stage, decisions were negotiated by healthcare professionals and family, and transfer delay benefitted patients that received more rehabilitation. During the stable stage, transfer decisions were made by non‐clinical staff as vocational nurses lacked the leadership to participate in discharge decisions. Transfer delay at this stage, ignoring patient readiness, was expensive and counterproductive.

Our findings were supported by a recent study on care transitions in patients with TBI showing that most transitions were appropriate with only 9% considered delayed or premature, mostly transfer out of ICU (Borgen et al., 2021). Premature transitions were assumed to reflect pressure to free acute‐care beds, while delayed transitions were characterized by heterogeneous patient trajectories, often related to waiting times for destination beds or to other non‐clinical care decisions (Borgen et al., 2021). This supported our findings that early in‐hospital or interhospital transfers were necessitated by new incoming patients. Our study showed that physicians tried to take the risks into account, although patient transfer could not always be avoided.

A study from the United Kingdom (UK) showed that critical care interhospital transfers were ‘time critical high‐risk’ episodes that often failed to meet the national standards (Grier et al., 2020). Some transfers were premature, as a UK study demonstrated that the intra‐hospital transfer of brain‐injured patients presented a potential risk, even when performed by skilled personnel (Picetti et al., 2013). Both American and European studies have shown that bouncebacks after transfer from a neuro‐ICU are common (Coughlin et al., 2018; Rhodes et al., 2012). Patient transfer has been determined by economy, using diagnosis‐related groups to decide which patients to transfer (Busse et al., 2011). Other incentive structures are suggested such as patient‐centred care because most healthcare systems are organized to treat single conditions rather than multimorbidity (Editorial, 2018). More recent studies suggest that family‐centred care during the acute stage might be more relevant, as patient transfer affects the whole family (Karlsson et al., 2020). Family caregivers were involved in decision‐making at the subacute stage in our study. In one of our examples, we cited a mother speaking on the patient's behalf, which delayed transfer (positively) and gave the patient an extended period of rehabilitation. As such, family involvement in transfer decisions could potentially help the patient and alleviate family caregivers' concerns.

During the subacute stage, less pressure was exercised in relation to patient transfer because the professionals were self‐determined regarding patients' length of stay. New admissions were negotiable. Patient transfer was determined over the course of weeks during highly structured treatment and team meetings with a high degree of collaboration and a flat hierarchy with family involvement as a strong facilitator. Extended stays were negotiable as professionals and families had shared authority.

The stages of the TBI‐trajectory were handled differently at the three sites as the patients' needs changed as their health improved. During the stable stage, patient needs were less medical and included more complex bio‐psycho‐social issues. At this point, the main daily caregivers were vocational nurses that were educated in the practical assistance of the residents with little medical knowledge. The complexity of the residents' needs increased the number of external collaborators and bureaucracy. We believe that the stable stage of the trajectory could be improved by including Registered Nurses as the daily staff to handle more complex issues and participate in clinical decision‐making and leadership. We found that the external collaborators were less dependable than in‐house staff and that lack of continuity led to information loss. An American review described similar issues as family caregivers experienced the final transition as fragmented and unsatisfactory for supporting a successful return home (Piccenna et al., 2016). The review suggested tailored education and patient and family involvement to increase readiness for returning home and reduce unplanned re‐admissions. In our study, the vocational nurses were concerned by the lack of engagement by GPs when a resident deteriorated. To alleviate this issue, a recent UK study recommended written plans to reassure the care home staff and to reduce their concerns (Harrad‐Hyde et al., 2021).

In the Danish context, the final transition requires residents and family caregivers to navigate between different healthcare sectors to attain necessary treatment after discharge to their home (Graff et al., 2018; Guldager et al., 2018; Willis et al., 2016). Unassisted, some patients were unable to take full advantage of the available healthcare services. Again, Registered Nurses prepared with a bachelor's degree and more education within clinical leadership, rather than vocational nurses, could improve assistance with healthcare services. Studies show that some patients that did not qualify for highly specialized rehabilitation chose to pay for private rehabilitation to expedite their recovery although free treatment was available (Graff et al., 2018; Egerod et al., 2020). Some patients, however, did not know what other treatment options were available because information was inadequate. Notwithstanding, one study shows that highly specialized rehabilitation was offered to 84% of eligible patients in Denmark (Odgaard et al., 2015), whereas only 31% get this offer in other European countries (Jacob et al., 2020). As such, the Danish situation is comparatively good.

Although the allocated daily cost for each stage in the trajectory was reduced, our study shows that the costliest stage per patient is the highly specialized rehabilitation during the subacute stage, where many specialists participate in treatment, and family caregivers are involved in decision‐making. We were unable to calculate the time spent or cost of transfers to interim units between sites A and B, but a recent Danish small‐scale study showed a mean length of stay in Neuro‐ICU of 17 days, at interim wards (detours) of 11 days, at highly specialized neurorehabilitation of 74 days and mean trajectory of 100 days for patients with TBI (Egerod et al., 2021). Consequences for patients were seen in mean duration of posttraumatic amnesia (PTA), which was 41 days for patients transferred to Site B still suffering PTA. A scoping review suggested that improved sleep could help resolve PTA, cognitive impairment and agitation during the subacute stage (Poulsen et al., 2020), but sleep was, perhaps, disrupted by multiple transfers and transitions, which could further impact the outcome.

Although an upgrade of the nursing staff at Site C would be more costly, we believe the length of stay could be substantially reduced by integrating Registered Nurses as in‐house clinical staff in the discharge meetings to enhance and expedite the final transfer to home or permanent care.

### 5.1 Limitations

Our study was enriched by fieldwork at multiple sites. Twenty‐one in‐situ interviews were conducted with a variety of professionals, painting a broad picture of the TBI treatment and rehabilitation trajectory and decision‐making related to patient transfer. Unfortunately, publication of our study was delayed by to the COVID19 epidemic and other pragmatic issues, but we assume the results would be much the same at this time as the context has not changed.

## 6 CONCLUSIONS

Transfer decisions were facilitated by expert physicians and assisted by nurses during the acute phase of the TBI‐trajectory. During highly specialized rehabilitation, transfer was facilitated by negotiation among in‐house healthcare professionals, community staff and family in a flat hierarchical structure. Transfer practice was less transparent during the final stage of communal rehabilitation as the complexity of issues and bureaucracy increased. Final transfer home was facilitated by vocational nurses based on patient‐centred values and practical knowledge of complex needs but inhibited by multiple external collaborators and less focus on clinical leadership. We recommend adding Registered Nurses with specialized education to the in‐house staff to improve clinical leadership and decision‐making within rehabilitation and the transition to the patient's home or permanent care. We also suggest more family involvement to support the patient and family values. More evidence is needed to identify the expertise needed at the final stage of this costly and time‐consuming trajectory.

### 6.1 Relevance to clinical practice

Nurses are an integral part of treatment and decision‐making in the TBI trajectory. Higher awareness of the facilitators and inhibitors of timely patient transfer should inform nurses and improve their decisional capacity. Knowledge sharing between the primary and secondary health sector is a communal facilitator.

## AUTHOR CONTRIBUTION

KH conducted the fieldwork, performed initial analysis and initial drafting of the manuscript. KH, IP and IE conceptualized the study and participated in the analysis and interpretation of data. IE drafted the manuscript and made revisions based on discussion with all authors, who approved the final manuscript.

## FUNDING INFORMATION

The authors used no external funding.

## CONFLICT OF INTEREST STATEMENT

The authors have no conflicts of interest to declare.

## ETHICAL APPROVAL

The study did not require Research Ethics Committee approval.

## PATIENT CONSENT STATEMENT

No patient consent was needed in this study.

## Data Availability

Data available on request from the authors. Data are in Danish.
